# Development of big data assisted effective enterprise resource planning framework for smart human resource management

**DOI:** 10.1371/journal.pone.0303297

**Published:** 2024-05-20

**Authors:** Yaxuan Zhao

**Affiliations:** Business School, University of International Business and Economics, Beijing, China; National Textile University, PAKISTAN

## Abstract

The planning of human resources and the management of enterprises consider the organization’s size, the amount of effort put into operations, and the level of productivity. Inefficient allocation of resources in organizations due to skill-task misalignment lowers production and operational efficiency. This study addresses organizations’ poor resource allocation and use, which reduces productivity and the efficiency of operations, and inefficiency may adversely impact company production and finances. This research aims to develop and assess a Placement-Assisted Resource Management Scheme (PRMS) to improve resource allocation and usage and businesses’ operational efficiency and productivity. PRMS uses expertise, business requirements, and processes that are driven by data to match resources with activities that align with their capabilities and require them to perform promptly. The proposed system PRMS outperforms existing approaches on various performance metrics at two distinct levels of operations and operating levels, with a success rate of 0.9328% and 0.9302%, minimal swapping ratios of 12.052% and 11.658%, smaller resource mitigation ratios of 4.098% and 4.815%, mean decision times of 5.414s and 4.976s, and data analysis counts of 6387 and 6335 Success and data analysis increase by 9.98% and 8.2%, respectively, with the proposed strategy. This technique cuts the switching ratio, resource mitigation, and decision time by 6.52%, 13.84%, and 8.49%. The study concluded that PRMS is a solid, productivity-focused corporate improvement method that optimizes the allocation of resources and meets business needs.

## 1. Introduction

Enterprises rapidly realize the importance of big data in today’s data-driven environment for streamlining their operations and decision-making procedures. Enterprise Resource Planning (ERP) and Human Resource Management (HRM) are key areas where big data has a lot of technology that can revolutionize how businesses manage their workforces, boost productivity, and promote economic growth when integrated with ERP systems [1]. The smart HRM system may intelligently assign resources to enterprises or departments following their appropriateness and abilities through analysis of information about human resources, including worker abilities, expertise, and productivity. This wise resource allocation boosts production and operational effectiveness [2]. Real-time feedback and tracking systems in HRM using big data technology by utilizing real-time HR data collection and analysis enables managers to quickly spot performance gaps, respond to employee requirements, and give timely feedback for development [3]. The technology can provide a more accurate and holistic evaluation of employee performance by analyzing full HR data and considering many elements outside typical measures. Technology can help smart recruitment and hiring, making objective and equitable performance appraisals easier. Effective HRM is essential for an organization’s success since it directly impacts worker efficiency, involvement, and overall business results. The manual and subjective approaches used in traditional HRM processes made it difficult to thoroughly understand employee abilities, achievement, and learning needs. However, big data technologies can potentially make HRM a more planned and data-focused function. The study aims to identify innovative approaches for streamlining HRM procedures, bettering resource allocation, and reaching greater efficiency and productivity by investigating how to incorporate big data technologies into the present ERP system [4].

The fundamental element of human capital is expertise and describe working expertise, specifically as the competence or knowledge developed via regular work practice. The importance of human capital is growing as many sectors and businesses move from labor-intensive to knowledge-intensive models. It is thought that building up human capital will result in company success and economic expansion [5]. To understand staffing, HR development, establishment, and overall enterprise operation, a full corporate development improvement necessitates an all-encompassing research and choice help model of the venture enhanced [6]. This study emphasizes how big data improves HRM through ERP systems, leveraging modern analytics for informed decision-making. It aims to integrate big data into ERP for simplified HR data management and assess its impact on HRM, productivity, and organizational success. HRM involves hiring, creating labor rules, and retaining them, which is essential for every company. Businesses use human resource management to keep employees secure and contented [7]. Enterprise resource planning (ERP) is a software module businesses use to connect the company with its employees better. ERP examines the specific pieces of information needed by businesses. ERP considers a company’s resources and uses this data to make informed decisions. ERP is a condition- and function-based business management system. Companies of any size may benefit from using an electronic HRM system. The primary goal of electronic human resource management (E-HRM) is to lessen the financial and time commitment involved in administrative tasks. Electronic human resource management (E-HRM) pinpoints the critical human resource-related components that provide superior data for downstream business operations. By providing incentives based on resource availability, E-HRM encourages workers to do quality work [8].

Human resource management (HRM) uses big data (BD) accumulation in businesses and organizations. Information extraction from large data sets is a popular use of big data. The big data accumulation approach shortens the time it takes for calculations to finish, expanding the effective scope of the systems. Information useful for forecasting in management systems may be accumulated using the accumulation approach [9]. Human resource management at the corporate level extensively uses big data to enhance management capabilities. Effective data required to complete a business task is predicted by BD [10]. BD advocates for a wide variety of high-quality HRM solutions in businesses. Database complexity is shown by the BD accumulation approach [11]. The accumulation approach simplifies the identification and calculation procedures involved in managing resources. The accumulation of BD is also employed in high-tech businesses to maximize returns on investments of scarce resources [12]. Data is gathered using the Accumulation technique, then analyzed using predetermined criteria. Increased accuracy in management is made possible by the availability of big data [13].

Big data analytics (BDA) is a method for extracting meaningful information from massive datasets. The widespread use of BDA has real-world consequences for certain industries [14]. Smart human resource management (SHRM) and utilization are two more business intelligence applications (BDA). Among BDA’s many applications is tracking database changes [15]. BDA reveals the technological tactics used by SHRM systems. BDA generates data that can be used to boost the effectiveness of a company’s human resource management and upkeep procedures [16]. BDA improves SHRM systems’ overall efficiency and dependability. In HRM systems, the BDA approach supported by AI is often employed [17].

The database is the primary location where AI is put to use. AI uses the feature selection approach to choose the characteristics and elements needed for organizational tasks [18]. Important SHRM strategies and methods are identified using AI. Using BDA powered by AI reduces calculation time, increasing system efficiency [19,20]. This article explores the prospects HR professionals have thanks to talent analytics. This paper compares talent analytics to other branches of business analytics and explores the advantages and disadvantages of using this strategy inside a company. Several examples of how talent analytics has helped a company make better choices will be shown. This research identifies critical pathways via which enterprise performance analytics might enhance the effectiveness of human resources departments and, by extension, whole businesses. This study addresses the costs (in terms of governance of data and ethics) that broad usage of talent analytics might produce while emphasizing the benefits that worker analytics brings to businesses. The significance of trust in ensuring the effective rollout of HR analytics initiatives. The study focuses on the difficulty of bettering business operations and human resource management. Manual and subjective HR practices prevent companies from maximizing the talents of their employees. This research proposes a Placement-Assisted Resource Management Scheme (PRMS) to increase organizational efficiency and financial performance by optimizing resource allocation using big data and transfer learning.

The novelty of the proposed work lies in the PRMS integrates big data analytics, transfer learning, and skill-based resource allocation, making it innovative to optimize resource management within enterprises. This novel strategy improves efficiency by matching resources to talent-based tasks, optimizing strategies using data-driven decision-making, and establishing exact management states for optimal business results. The PRMS improves performance indicators over existing methods, giving it a credible platform for improving company efficiency and financial results.

The proposed system is motivated to enhance business operations and financial outcomes through efficient resource management. Optimizing resource placement and utilization is essential for producing better results because HR planning and management of enterprises are critical to an organization’s success. This research attempts to provide an effective and productivity-focused resource allocation strategy by utilizing knowledge, organizational requirements, and a data-driven approach. The objective is to improve enterprise operations, allocate resources efficiently, boost efficiency, and produce better financial results, eventually enhancing organizations’ competitive edge and growth. This research proposes an innovative HRM and business growth method by utilizing the existing ERP system to incorporate big data technologies, machine learning, and transfer learning. The novel aspect is the implementation of a Placement-Assisted Resource Management Scheme (PRMS), which optimizes resource allocation by employee talents to boost output and efficiency. PRMS uses trial periods to assess resources and historical data to position them in roles that best utilize their skill sets. Adaptive resource allocation made possible by transfer learning boosts business performance. The new aspect of this research is its holistic approach to resource management. This can help businesses greatly by streamlining HR procedures and increasing efficiency. The article’s contribution is summarized in the points below:

1. Designing a placement-assisted human resource placement and management scheme (PRMS) for improving the enterprise’s financial operations.
2. The proposed method utilizes resource swapping and skill utilization/ update for enterprise-oriented results through a precise management state.
3. The PRMS incorporates the last known enterprise’s successful operation for training, sustaining/ leveraging enterprise operation profitably.
4. The experimental results show a better performance for data analysis with a suitable source and strengthen the proposed scheme’s liability.

The upcoming sections in this paper are as follows: section 2 examines the related work; Section 3 determines the enterprise’s performance and improves the enterprise management; Section 4 demonstrates the results and discussion; Section 5 concludes the overall paperwork.

## 2. Related works

Chen et al. [21] developed a new human resource management (HRM) system using B/S mode. The actual goal of an HRM system is to analyze the system function, activities, roles, and non-function of an application. Both small and medium-sized enterprises contain HRM, which reduces the complexity of further processes. The exact aspects or content of the function are identified, producing appropriate HRM data. The B/S mode improves the overall performance level of HRM systems. When there are some inevitable problems in the system, the system can be easily repaired. For example, the database can be automatically backed up and restored to avoid the loss caused by system damage to the enterprise. Additionally, the section only discussed the system’s economic advantages without taking other crucial aspects of HRM into account, like staff involvement, talent development, and organizational culture.

Liu et al. [22] introduced an FPGA and data mining-based human resource management (HRM) platform for enterprises. The data mining approach provides control strategies that detect the relevant data for management systems. Multi-dimensional variables and attributes are analyzed using the FPGA model, reducing the computation process’s latency. Spatial and temporal features are also detected from the database that produces relevant data for management processes. Experimental results show that the introduced platform maximizes the efficiency and reliability range of the systems. However, data similarities and differences are not identified, and the text also omitted information on the precise variables or sources used to evaluate and manipulate HR data.

Jian et al. [23] proposed a data mining-based human resource decision support system for enterprises. The main aim of the proposed method is to support decision-making by providing appropriate human resources. Data mining technique predicts the exact data which are required for decision-making processes. The proposed method achieves high accuracy in the decision-making process. The proposed method improves an organization’s effectiveness and performance level of human resource management. However, generating a classifier quickly and effectively is a big problem for a given data set. One weakness of the study is the absence of particular information regarding the design and implementation of the business HR decision support system based on data mining.

Ahlemann et al. [24] designed a resource-based theory (RBT) human resource management system for enterprise architecture management (EAM). The RBT is mainly used here to understand the exact need for a function or process. As a result, RBT reduces both time and energy-consuming ranges in the computation process. Furthermore, RBT identifies the necessary data required for EAM benefits that reduce the computational cost of the systems. As a result, EAM creates effective benefits and functionalities that enhance the performance range of small enterprises. However, the study relies primarily on interview data and document analysis. Considering the sample size restrictions, theoretical viewpoint, lack of specific practical advice, and alternative suggestions are important.

Jaouadi et al. [25] proposed a big data analytics-based human resource management method for organizations. The goal is to innovate the supply chain, providing relevant ideas for the organizations. Big data analytics is mainly used here to detect the exact capability level of data presented in the database. The proposed method creates an integrated platform to maintain the human resources in the organizations. However, the impact of BDA in achieving business performance is not substantial due to the lack of firm technological capabilities. The report makes recommendations for politicians, but it doesn’t offer any guidance or ideas on how to deal with the elements it has found.

Liu et al. [26] developed a data-driven analysis of employees’ enterprise expertise. The random forest (RF) model is used here to identify the excellent talent among the employees. RF is a machine learning (ML) model used here to achieve high accuracy in detection and prediction processes. RF model produces the exact data for human resource management systems. The actual aim of the proposed method is to improve the performance range of employees in enterprises. Additionally, there may be restrictions on the data used for training and generalizability to diverse employee groups for the machine learning model presented for forecasting development potential.

Table 1 presents the summary of references that deals with enterprise development. However, supervised learning classes in the data are imbalanced.

**Table 1:** Summary of references for enterprise development.

Author name	Key area	Technique	Limitations	Results
He et al. [27]	Create effective communication services among employees and organizations.	The main aim is to create an impact on HRM systems.	The comprehension of how these technical uses are put into effect in different departments of HRM is further constrained by the lack of concrete examples	Increases the benefit level of the enterprises.
Zhang et al. [28]	The proposed method is a fuzzy-based human resource management strategy that reduces management systems’ complexity.	The butter Ant optimization (BAO) technique is also used here to improve the performance range of enterprises.	Not enough attention is paid to other crucial factors, including affordability, environmental sustainability, and consumer happiness.	Maximizes the efficiency and reliability range of the enterprises.
Tamym et al. [29]	The actual goal is to maximize the sustainability level of NVs.	BD is mainly used here to analyze the relevant data for the human resource management process.	Without considering other crucial elements for long-term sustainability, such as company governance, involvement of stakeholders, and ethical procedures, the paper concentrates on the adaptability and robustness of NEs.	Increases the robustness and flexibility range of NVs.
Novikov et al. [30]	It is a method that predicts enterprises’ exact production and manufacturing range.	ML algorithm is used, which detects the relevant data for the management process.	The study does not cover the degree to which the suggested methodologies can be used in various contexts, such as industries, business sizes, and production processes.	Increases the accuracy of decision-making processes.
Tamym et al. [31]	Create an effective platform to improve the production level of networked manufacturing enterprises.	BDA identifies the optimal data for manufacturing enterprises.	The paper does not address the issues of comprehending and presenting the results produced by the BDA process, which instead emphasizes the additional benefit of the suggested technique.	Improves the efficiency and feasibility range of the enterprises.
Ye et al. [32]	It provides optimal strategies for planning the schedules for enterprises.	The deep reinforcement learning (DRL) algorithm is implemented in IPS, which schedules the plans.	The restrictions on data accessibility and quality may impact the efficacy and generalization of the suggested methods.	Reduces the latency in planning processes.
Freire et al. [33]	The main aim is to improve the accuracy of scheduling.	Use appropriate data which are presented in the database.	The practical applicability and efficacy of the suggested ranking and mathematical framework are still unknown without empirical validation	Reduces the maintenance cost and energy consumption range in the computation process.
Sislian, L., & Jaegler, A. [34]	The article aims to improve ERP modules using blockchain technology to achieve better financial results.	To investigate how the various ERP modules interact with one another and affect business finances.	While the article makes recommendations for businesses, it does not detail the difficulties and practical challenges associated with executing ERP and blockchain connectivity.	It provides a fresh understanding of blockchain technology and the opportunity to work with ERP to boost financial results.
Chowdhury, S. et al. [35]	This paper systematically reviews multi-disciplinary literature from global business, Management of Information, Operations Administration, General Management, and HRM to provide an exhaustive and objective understanding of the organizational resources needed to develop AI capability in HRM.	To objectively self-assess organizational preparedness and build plans to adopt and deploy AI-enabled HRM practices and procedures.	The study listed several organizational resources crucial for the widespread use of AI in HRM; however, it offers no recommendations on allocating or prioritizing resources properly	The AI capabilities framework integrates resource- and knowledge-based view theories to identify organizational resources needed for business advantages.

Yao and Masoumeh [36] examined how the productivity of human resources in the COVID-19 age is impacted by employee skills and knowledge, company economic conditions, present IT infrastructure, payment trends, cloud availability, and cloud privacy and security. Stress, a large labor force, decreased organizational performance and profits, unfavorable organizational conditions, an inability to manage, and a lack of training can all impact the productivity of human resources during COVID-19. As a result, this study aims to examine how productive human resources are in the COVID-19 period. Based on the results, HR efficiency in COVID-19 is positively impacted by employee skills and knowledge, company economic conditions, payment trends, cloud accessibility, and the organization’s current IT infrastructures. The limitations identified are no controlled interventions or experimental manipulations of variables, which may restrict the capacity to draw firm conclusions regarding causal linkages and the efficacy of possible therapies.

Alsharari [37] evaluated the key elements that made the ERP system successful, how to apply it at Musanada Corporation in the United Arab Emirates, and any employee opposition to this system. This study uses a qualitative methodology and gathers data from company stakeholders (company members). A thorough evaluation of the submitted research and ideas for system development. The field study within the analysis steps during the execution and application of the system in the government bodies of the United Arab Emirates is provided in this paper, along with the results entered by the group members and through gathering information from stakeholders for the organization. The result findings involve planning, designing, development, training, live, and evaluation performance. The study’s breadth may be constrained by a lack of time or resources for data collection and analysis, which could hinder an exhaustive review of all pertinent elements.

The absence of research or tactics primarily geared towards examining how human resource planning affects achieving organizational objectives, especially in small and medium-sized firms. The study that is needed to explore and pinpoint the precise ways that human resource planning might assist organizations in overcoming employee weaknesses and achieving their goals is where the gap is. This study aims to create a Resource Management Scheme (PRMS) with an emphasis on productivity and examine the effect of big data on human resource management (HRM) in organizational settings. The study’s stated objectives are to develop a unified strategy for managing resources, enhance resource allocation, and assess how key performance metrics, including efficiency, staff satisfaction, and organizational effectiveness, are affected by HRM that is enabled by big data. The framework will allow more effective HRM practices by incorporating significant data insights into the current Enterprise Resource Planning (ERP) system. Resource allocation inside the PRMS will be skill- and operation-based, boosting efficiency and effectiveness. The influence of extensive data-enabled human resource management on KPIs is an essential area of study, as is the optimal strategy for incorporating big data technology into an existing enterprise resource planning (ERP) system.

Methods for optimizing HRM systems and businesses as a whole are discussed in the literature. Some research emphasizes efficiency and financial gains, while others overlook the significance of factors like employee motivation and professional growth. While others have proposed an HRM platform built on FPGAs and data mining, they have yet to demonstrate a thorough familiarity with the commonalities and distinctions present in HR data. While a data mining-based HR decision support system might boost productivity, some obstacles still exist to developing classifiers. While resource-based theory (RBT) human resource management (HRM) is effective, it does call for additional hands-on guidance. Although big data analytics has been used, reliable technological capabilities still need to be improved. Employee expertise is analyzed using a random forest model, which has problems with data restrictions and generalizability. The proposed technique should evaluate HRM systems by considering employee participation, data variation, fast classifier creation, RBT implementation, boosting technological capacities, and resolving data restrictions to make accurate predictions. Our suggested technique for ERP in HR is aimed directly at HR tasks, including educating personnel, handling payroll, and monitoring worker progress. As a result, it significantly reduces the work required of a human resources manager.

Existing research emphasizes the importance of using big data, machine learning, and optimization strategies to enhance HRM procedures and business output. However, there are voids in ensuring things like cost-effectiveness, environmental responsibility, and customer happiness. To fill in these blanks, we suggest the Placement-Assisted Resource Management Scheme (PRMS), which combines knowledge, business needs, and data-driven approaches to boost productivity, efficiency, and profitability. This all-encompassing method fills in the blanks left by previous studies and offers a workable answer to businesses that want to boost their HRM and productivity.

Human resource management systems (HRMS) modules are added to ERP systems for further HR functionality. Modules like this aid with people-focused tasks like recruiting and administration. They streamline internal operations by computerizing formerly manual procedures. Unlike ERP, a fully integrated human resource management system (HRMS) has many useful features. Besides, the previous enterprise solutions for sustainable improvements are considered for leveraging the current state. In this assessment, the previous enterprise outcomes are correlated with the human resource skills and performance contribution towards the enterprise developments. Therefore, this scheme is reliable in balancing other features for better outcomes for a sustainable period.

### Introduction to enterprise development challenges

Enterprise development success depends on optimizing HRM processes. However, traditional approaches to HRM typically fall short of addressing critical factors such as worker involvement, talent development, and corporate culture. Some HRM systems, like B/S mode or FPGA and data mining platforms, offer economic benefits but may overlook these significant factors.

### Identifying gaps in existing approaches

One standard limitation of existing HRM systems is their focus solely on economic benefits, neglecting other vital aspects of HRM. For instance, while data mining-based decision support systems show a premise in improving decision-making processes, challenges persist in rapidly generating effective classifiers. Similarly, RBT-assisted HRM systems lack practical guidance for implementation, and big data analytics-based methods face limitations due to technological capabilities and neglect crucial aspects like staff involvement and talent development.

### Introduction of PRMS with its key features

The research introduces the PRMS; unlike traditional models, it integrates knowledge, business needs, and data-driven methodologies to address these gaps and complexities and enhance enterprise productivity, efficiency, and profitability. By prioritizing factors like staff involvement and talent development alongside economic benefits, PRMS offers a holistic solution for sustainable improvements in enterprise development.

PMRS represents a significant advancement in HRM practices, offering a holistic approach beyond economic benefits to address critical aspects of enterprise development. By leveraging previous methodologies and incorporating key features like HRMS modules into ERP systems, PMRS aims to drive sustainable organizational performance and success improvements.

While similar methodologies have been implemented to address unrelated problems, such as data mining-based DSS in healthcare or resource-based theory in organizational management, they share common challenges with traditional HRM approaches. These challenges include overlooking critical factors like staff involvement and talent development in favor of economic benefits. Therefore, the introduction of PMRS as a comprehensive solution underscores the need to prioritize these factors for sustainable improvement across various domains.

## 3. Placement-assisted resource management scheme

### Problem statement

Many proprietors of small and medium-sized businesses lack strategies to use information technology to streamline processes and keep the focus on core company competencies. Because accounting systems offer only accurate data about company situations, the need for an analysis perspective has been felt at the top management level of several companies. Human resource planning, as an essential variable, can be used to remove employee deficiencies in an organization. As a result, the organization will achieve its aims and objectives if human resource planning is appropriately done. However, the primary interest of this study is the impact of human resource planning on attaining an organization’s goals.

The research aims to create a Placement-Assisted Resource Management Scheme (PRMS) that optimizes organizational resource allocation using big data analytics and transfer learning. The main goals are to improve business operations and financial results through efficient resource management, skill matching, and precise management states for improved business results. Resource management tactics use big data and transfer learning to improve decision-making and operational efficiency. The study also examines how skill-based resource allocation affects organizational productivity and identifies essential performance measures to assess the PRMS’s impact on resource management and financial results. These inquiries and goals attempt to provide a complete framework for effective utilization of resources and increased company performance in various types of organizations.

The objective of effectively managing resources according to their talents and enterprise requirements is to increase productivity and produce effective business successes using the proposed PRMS. The novelty of the research idea concentrates on enhancing business processes and financial results through efficient planning of resources and utilization with the help of the proposed PRMS by using data-driven insights to improve resource management and decision-making. The approach seeks to maximize skill-based resource allocation. Transfer learning intends to continuously enhance resource management tactics by drawing on trial outcomes, profitable company ventures, and resource performance data. The significance lies in enhancing business operations and financial results through efficient resource placement and utilization that takes advantage of big data technology and skill matching.

Human resource planning is the continuous procedure of methodological scheme to use an organization’s most peerless benefit caliber employees perfectly. Human resources planning secures the outstanding apropos between employees and jobs while eliminating workforce scarcity or supernumerary. Human Resources is the subdivision within an organization liable for maintaining its employees. HR is responsible for hiring, firing, and managing employee benefits and other employees’ needs. Corporations’ human resource planning and management consider their proportion, workable efforts, and effectiveness for better enhancements. Resource placement and utilization are the preponderant conventionalities for enhancing enterprise procedures and pecuniary outages. It is the lurking factor for enterprise growth based on efficaciousness and expedition. This article proposed a Placement-Assisted Resource Management Scheme (PRMS) for productivity-focused enterprise improvement. The available resources are substantiated by their knowledge and work perfectness after the trial period.

This research takes a mixed-methods approach to examine how PRMS affects organizational performance and the happiness of its workers. Quantitative information is gathered via standardized surveys and tests, whereas qualitative information is gathered via semi-structured interviews and open-ended questionnaires. Using a stratified sampling technique helps businesses accurately reflect their diverse employee and customer bases. Triangulation of data, testing for dependability between raters, analyses of test-retest consistency, and content validity all contribute to validating data. Human resource management (HRM), enterprise resource planning (ERP), and data analytics specialists review and modify the study approach to meet standards. The research process leaves a paper trail that includes notes, transcripts, data analysis stages, and any changes made to the original data. All participants consent after being fully informed, and their personal information is kept private and secure out of respect for their confidentiality. The reliability of interpretations is increased, and complete comprehension of the study challenge is ensured through member verification and peer debriefing sessions.

### Transfer learning (TL)

Transfer learning (TL) is a research problem in machine learning that aims to store knowledge acquired during resolving a problem and implementing it to a different but related problem. From this standpoint, reusing or transferring information from previously learned tasks to learn new tasks has the quiescent to enhance the sample efficacy of a learning agent. The proposed PRMS uses transfer learning to apply knowledge and insights learned from previous employment or projects to new allocation of resources scenarios. The learning system is trained using trial outcomes and prosperous company accomplishments through transfer learning. The learning system can better grasp the use of resources, patterns, and tactics that have produced beneficial results by applying knowledge from prior experiences. As a result, a precise management condition that can serve as the foundation for the best possible resource allocation is created.

### Use of big data in PRMS

Big data anatomization helps organizations utilize their data and use it to determine new occasions. Our suggested technique analyzes the enterprise’s performance and contributes to value creation by facilitating the exploitation of individual-level data and the development of measures that may individually assist personnel. Large amounts of financial accounting data the firm produces may be included in big data. Financial accounting data in PRMS can be found in structured and unstructured formats, including text documents, emails, financial statements, and transaction records. It is critical to manage and analyze this variety of data to manage resources and decision-making. This integration in PRMS may involve combining financial accounting data from various departments or systems to provide a complete picture of resource needs and performance. Significant amounts of accounting and financial information can be processed and stored effectively using PRMS by utilizing these features, providing rapid access and analysis, and managing resources.

**Fig 1:**
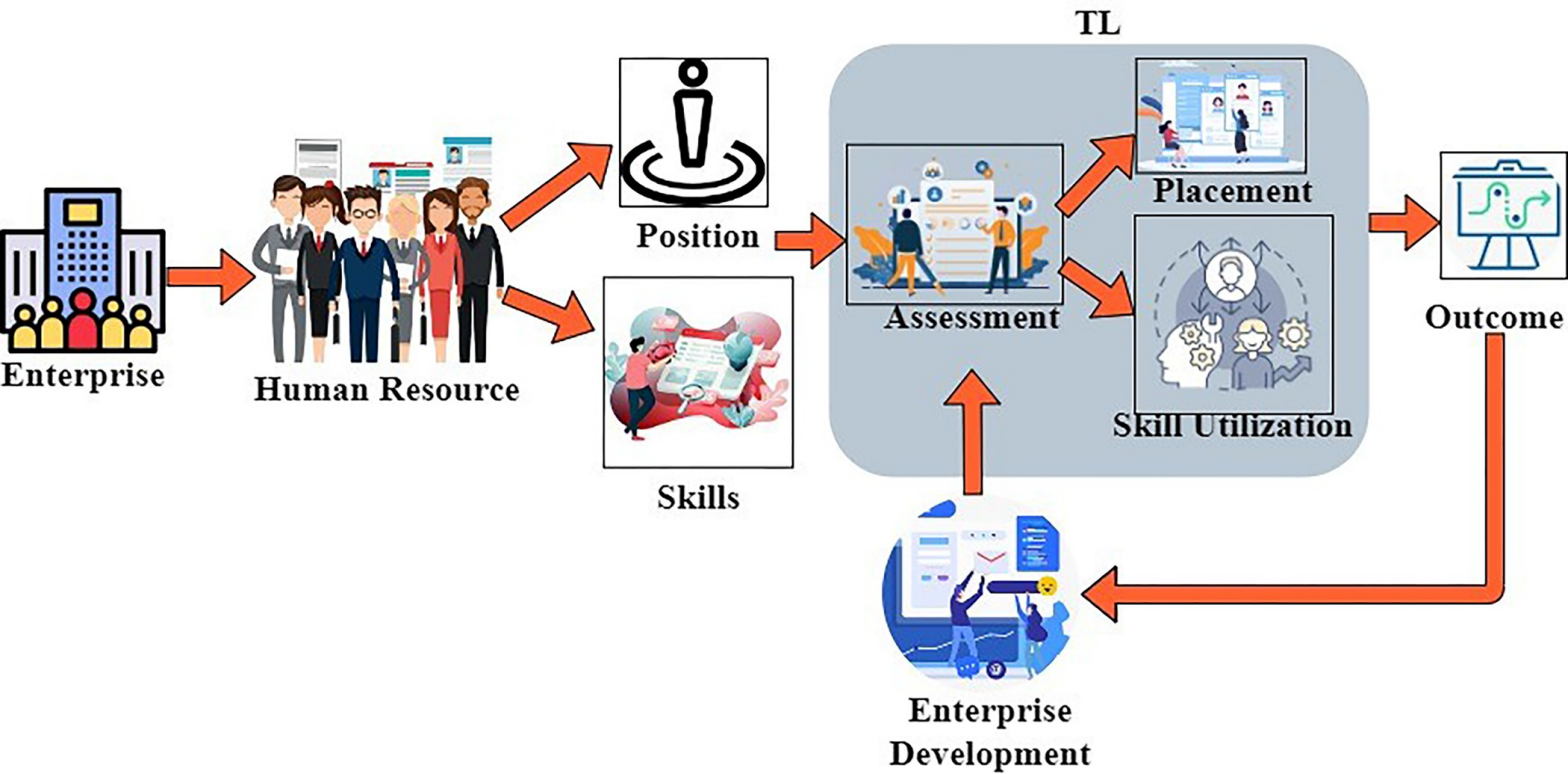
Proposed scheme.

This issue may be avoided, and the advantages of training can be identified by upper management, provided detailed information on individual performance is gathered. That, in turn, leads to smarter business moves, more efficacious procedures, and better profits. Businesses that use big data with advanced investigation acquire value in many ways. Fig 1 portrays the proposed scheme.

As per in Fig 1, an enterprises use human resource management to process operations on time and determine the mentioned position and the skills of the human resources. In the position determination, it acquires the HR designation, and in the skill determination, it identifies the skills executed by the HR, which leads to the effective maximum outputs. From this, the assessment process is done with the acquired data from human resources to verify the placement and the skill utilization for the perfect outcome. In the TL block, the assessment from the data gained is used to determine the positional/managerial placement of resources by pursuing their professional skills. The above-stated features, apt enterprise operations, and financial outcomes are verified first in an experimental manner by resource placement. Based on the above outputs, transfer learning is trained for identifying a correct management state. The states are modeled for resource placement and utilization individually for enhancing-enterprise results. The states are unchangeable and are trained using the resource performance at every level of the procedures.

Based on the important enhancements, the learning is trained using the last known advantageous enterprise achievement. This scheme is reliable in swapping resources based on skills and enterprise demands before relieving them from the enterprise position. In this method, the enterprise uses human resource management for the execution of the process on time without any delay. It is also used to check the position of the resources and also their skills in their work time. This management is used to find the operation procedure, whether it is processing at its correct time or not. It is also responsible for managing the employees to enhance their skills and knowledge about the enterprises. The processes processed at the correct time are stored in the management, and the human resource management maintains the employees. It organizes the benefits of managing the employees to deliver the process on time. Also, it will help determine the employers’ position and skills and whether it suits their vocabulary. The benefits of the proposed based ERP in HR as mentioned below,

- Facilitates the administration of rules and coordination among workers. It is an element of database management that helps store and manage the individual data of an organization’s personnel.
- It’s useful for all four pillars of management: strategy, operations, and leadership. To that purpose, it backs up any resources that could be used. Employee planning is an integral part of organizational management.
- It aids in managing a company’s financial records and provides insight into workers’ pay, benefits, and deductions. It also signals forthcoming alterations.
- ERP in HR allows for creating KPI reports for the whole company and information on individual workers’ performance across all divisions.

The process of utilizing human resources management in identifying whether the process is processed on time of the enterprise is explained by the following Eq (1) given below:

$$\begin{array}{c} ƞ(\alpha ,\varphi ,\varphi^{\left(1\right)},\ldots ,\varphi^{\left(n-1\right)})=\varphi^{(n),}\\ where\;\varphi^{\left(i\right)}=\frac{d^{i}\varphi }{d\alpha^{i}}\\ \hat{A_{ƞ}}\varphi =ƞ(\alpha );\hat{A_{ƞ}}\varphi =\sum_{i=0}^{n}\alpha_{i}(\alpha )\varphi^{\left(i\right)} \end{array}\}$$ (1)

**Fig 2:**
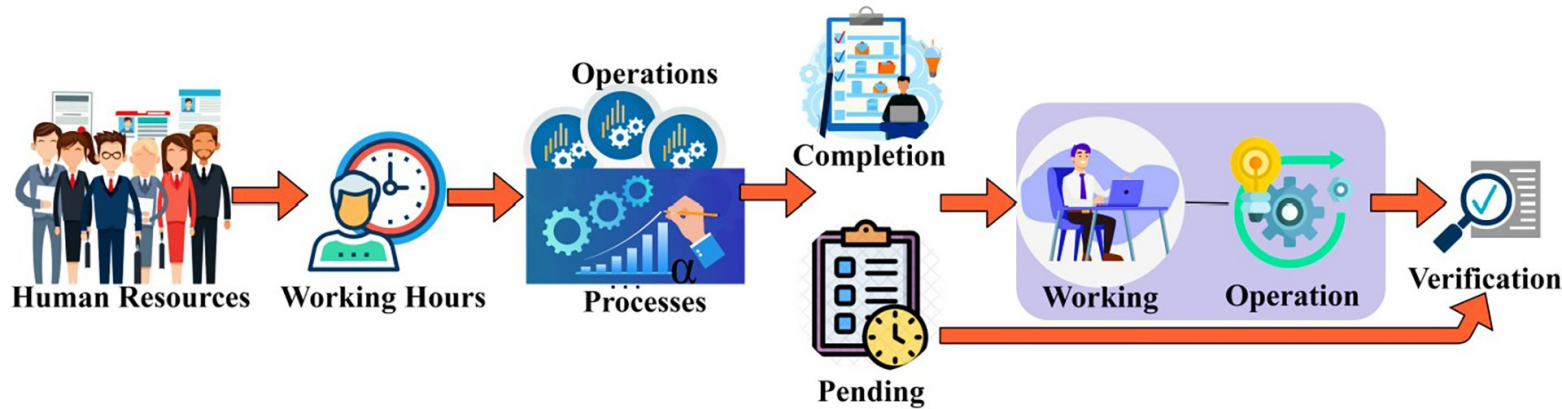
Operation time verification.

Where ƞ is denoted as the management of the human resources in the enterprises, *φ* is denoted as the verification process of the procedure, *α* is denoted as the process done by the human resource management, *A* is denoted as the verification process of checking the time taken to process the operation in the enterprises by the human resources. Now human resource management identifies the HR designation and their skills according to it. The enterprise’s management determines the employee’s position to estimate their working efficiency. It is used to verify whether the employees can process the operation on time in the enterprise with effective outcomes. It is also used to determine the skills of the acquired position of an employee. It checks whether the resource has the prompt skills to execute effective outcomes without delaying the processing of the operation. It is also used to estimate whether the employee has the skills according to their position in the enterprise, providing the maximum outcomes without any issues in the process. The employees’ skills should result in the maximum outcome during the processing time in the enterprises. The skills should help the employees deliver the operation on time with positive outcomes without any problems. Human resources management is used to identify the position of the resources that they can sustain for a longer time by providing an effective outcome with their outstanding skills. The operation time verification flow is represented in Fig 2.

The available human resources are allocated to different processes at different management levels. The operations/process are either completed or left out within working hours. Therefore the *α* is carried forward for verification by identifying their skills and position (Fig 2). The skills identified must fit the employees’ position. Then, only the skills will help accomplish efficient results with the maximum output without any issues. The procedure of delivering the process on time without delay is based on the position and the skills of the employees in the enterprises. Based on the working domain and operation features of the employee, the time of completion is identified in the next process. From the data acquired, the assessment procedure is done to identify the placement and the skill utilization with the help of the transfer learning technique. The output of the skills used by the employees based on the position to provide the maximum outcome will also be helpful in the determination of the skill utilization process in the assessment operation by the learning technique. The process of acquiring the position and the skills of the employees according to the position by the resource management in the enterprise is explained by the following Eqs (2) and (3) given below:

$$G=(\hat{A_{n}}\varphi_{\sigma }(\alpha )-ƞ(\alpha ){)}^{2}+(\bar{A_{0}}\varphi_{\sigma }(\alpha )-\varphi_{i}{)}^{2}$$ (2)

$$\begin{array}{c} L=L_{1}+L_{2}=(\hat{A_{ƞ}}H-\alpha^{1}(ƞ){)}^{2}+(\bar{A_{0}}H-\varphi_{i}^{1}{)}^{2}\\ \frac{\partial L}{\partial H}=(A_{ƞ}^{1}H{)}^{\alpha }(A_{ƞ}^{1}H-\alpha^{1}(ƞ))\\ \frac{\partial L_{1}}{\partial L_{2}}=(\bar{A_{0}}H{)}^{T}(\bar{A_{0}}H-\varphi_{i}^{1}) \end{array}\}$$ (3)

Where *G* is denoted as the process of identifying the position of an employee, *L* is denoted as the skills identified, *H* is denoted as the output produced by the resources with the identified skills. Then the assessment is done with the help of the transfer learning technique to determine the placement and skill utilization. The assessment procedure is processed with the above-stated data acquired from resource management. After the trial period, the available resources are identified for their skills and work perfectness. The transfer learning determines the HR skills gained during the trial period and, thus, the maximum outputs the resources perform. The assessments are used to verify the skills of the resources gained after the trial period in the enterprises. The efficiency of HR is estimated with the output of the assessment process and the employees’ performance. From that, the efficaciousness of the trial period and the skills of the resources are identified during the assessment process. Also, the state and the utilization of the skills by the HR are determined. The enterprise’s efficiency in providing the process on time is used in determining the output of the assessment procedure. The assessment process executes the HR performance with the acquired data from the enterprise and uses the identified skills to produce the maximum outputs without any delay or issues.

The assessment process uses the transfer learning technique, which helped store the gained knowledge in the trial periods and check the employee’s efficiency with the skills according to it. From the knowledge acquired from the trial period, the production of an effective outcome is done. The resources are used to resolve further problems during the processing procedure while producing effective outcomes using the acquired knowledge; the placement of the enterprise resources can be found in the output of the assessment process. Also, the skills used to deliver the productive outcome will be identified. The learning technique determines the knowledge of the resources and the skills in the assessment process. The process of assessment procedure with the data acquired with the help of the transfer learning technique is explained by the following Eq (4) given below:

$$\begin{array}{c} W_{\pi }=({\hat{A}}_{H}^{T}{\hat{A}}_{H}+{\bar{A}}_{H}^{T}{\bar{A}}_{H}{)}^{-1}(A_{H}^{T}{ƞ}^{1}(\alpha )+{\bar{A}}_{H}^{T}\varphi_{i}^{1})\\ \left(A^{\alpha }+A^{x}+A^{x\alpha }+V(\alpha ,x))\varphi (x,\alpha )=ƞ(x,\alpha \right) \end{array}\}$$ (4)

**Fig 3:**
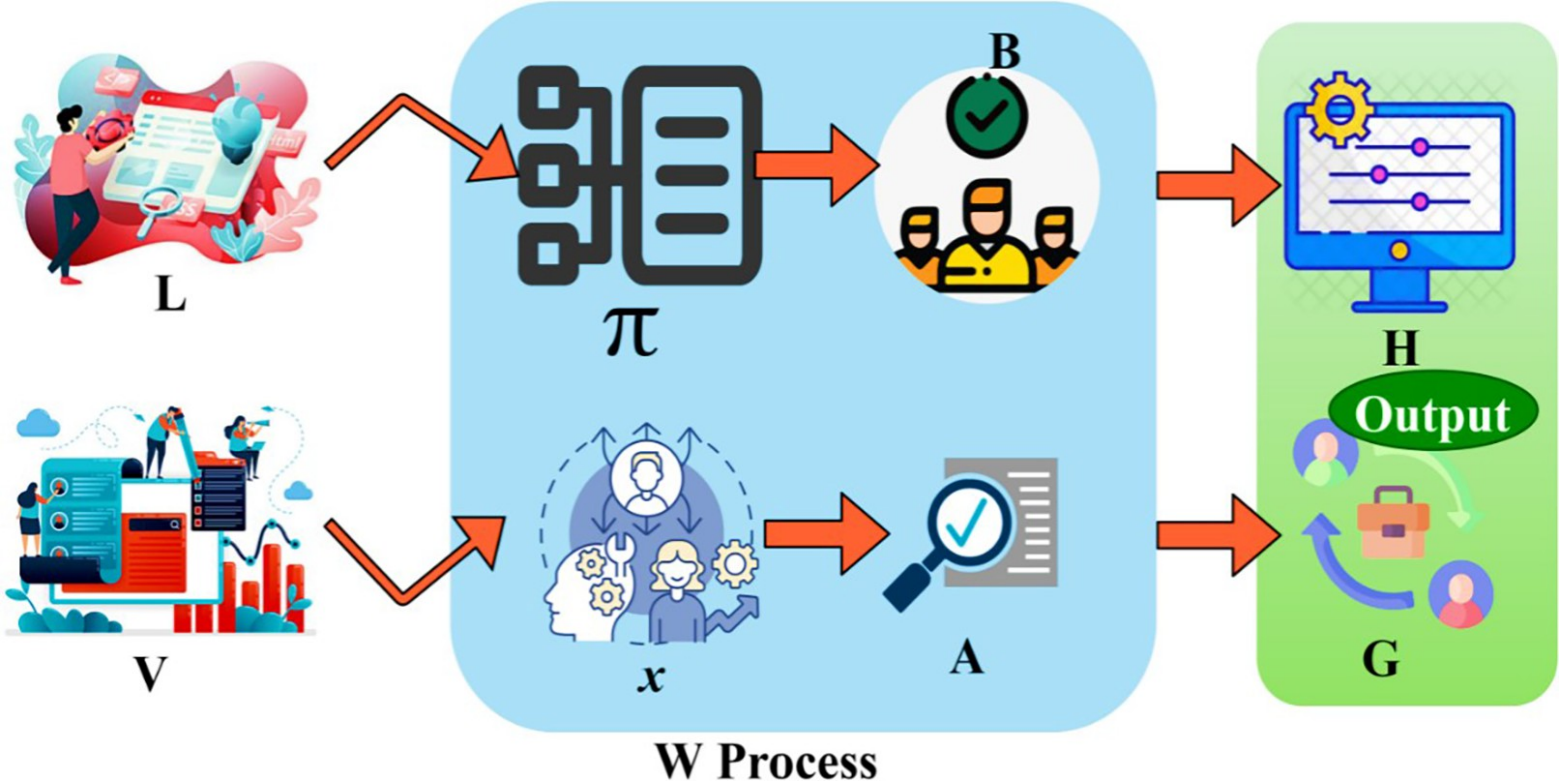
Assessment process using TL.

Where *W* is denoted as the process of the assessment procedure, *π* is denoted as the data acquired from the previous process, *V* is denoted as the outcome of the previous estimation of the position and skills process, *x* is denoted as the uses of the transfer learning technique in the assessment procedure. Now from the output of the assessment process, the placement is identified to produce an effective outcome. The factors mentioned above, such as perfect enterprise procedures and financial outputs, are determined first in a trial period by resource placement. The transfer learning is trained to identify a correct management state depending on the trial assessment outputs. This placement is used to identify the state of the resource management in processing the operations on time. This procedure can determine the output of the assessment and train the transfer learning to determine the perfect resource management state. The states determined by the transfer learning are modeled for the placement of the resources and utilization individually for enhancing the outcomes of the resources. The state of the management is used to determine the process level and whether it can deliver on time. The first assessment using TL is illustrated in Fig 3.

The representation for assessment (W) using transfer learning is presented in Fig 3. The *L* and *V* are the inputs in this learning process for which data acquirement and utilization are observed. If the *π* is sufficient, then *x* is carried forward for further verification. IF *B* is the only output, then *H* is the augmented skill along the existing *L*. Contrarily, if *A* fails, then *G* refers to the position/ employee level changes within the management. It is performed as a part of enterprise HRM. The output of the assessment is used to check the placement and the utilization of the skills with the help of the transfer learning technique. This learning technique is trained to provide the precise state of resource management.

From that precise management state, further improvements are made to the position and skills of an employee to provide an effective enterprise outcome. The output of the assessment is used to determine the placement of the resource management with the transfer learning technique. And this placement output and the skill utilization output are interconnected to provide an efficient outcome for the enterprises. From this, the recommendations will be given to enhance the assessment output if there is no efficacy in the enterprise outcome. The output of the position and skill identification process will be helpful in the determination of the placement and the skill utilization process. The data which is acquired from human resource management will be helpful in the assessment process. Thus, the output of the performance of the resources will be gainful in achieving the best enterprise outcomes. It is achieved by the most precise state acquired by the placement procedure with the help of the transfer learning technique. The states are unchangeable and are trained using resource performance at every process level. The process of determining the state of the management resources with the help of the learning technique in the placement procedure is explained by the following Eqs (5) and (6) given below:

$$\begin{array}{c} B=B_{I}+B_{H}+B_{\alpha }\\ (\bar{A_{\varphi }}-\alpha (ƞ,x){)}^{2}+(\varphi (o,x)-\alpha (x){)}^{2}\\ =\sum_{\pi =L,H}(\varphi (\alpha ,\pi )-B_{\pi }(\alpha ){)}^{2} \end{array}\}$$ (5)

$$\begin{array}{c} \frac{\partial L_{1}}{\partial L_{2}}=2\hat{A_{H}^{T}(\hat{A_{H}}W-}\alpha (ƞ,x)\\ \frac{\partial L_{I}}{\partial W}=2H_{o}^{T}(H_{o}W-\alpha (0,x))\\ \frac{\partial L_{B}}{\partial ƞ}=2H_{o}^{T}(H_{\pi }W-B_{\pi }(ƞ)) \end{array}\}$$ (6)

Where *B* is denoted as the process of identifying the management state, now the identification of the skill utilization process is done using the output of the assessment process. It determines the usage of the skills during the implementation of the effective outcome of the enterprise. With the help of the assessment process, the performance of the resources will be identified, and thus, some of the steps will be taken to enhance the resource performance, and thus it will be used in the development of the outcome. Skills utilization is used to determine the efficaciousness of the trial period done by the enterprise resource management. Big data is used to determine the state of the management, and therefore, the skills will be used to provide an effective enterprise outcome without any loss. The state of the management is unchangeable, and the performance of the resources in each process will train it. The utilization of management skills also determines the trial period’s efficiency. Skill utilization is identified from the output of the assessment process and the placement of the enterprise management. Then these two outputs are interconnected to determine the achievement of the enterprise outcome. Then the recommendations will be given to developing the enterprise if there is any loss in the outputs. The process of identifying the placement and the skill utilization is done by using the transfer learning technique with the help of the assessment process output. Then the transfer learning is trained to determine the precise state of the enterprise management to identify effective outcomes. Then the performance of the enterprise management is used to determine the skills they gained in the trial period. The process of the identification of the skills utilized by the enterprise management for the production of an effective outcome is explained by the following Eqs (7) and (8) given below:

$$\begin{array}{c} W=({\hat{A}}_{H}^{T}{\hat{A}}_{H}+\sum_{=O,L,W}H_{\pi }^{T}H_{\pi }{)}^{-1}\\ \begin{array}{c} =({\hat{A}}_{H}^{T}ƞ(h,\alpha )+\sum_{=O,L,W}H_{\pi }^{T}Q_{\pi }(\alpha ,x))\\ where\;Q_{\pi }=W(x),Q_{L}=B_{L}(\alpha ),Q_{W}=B_{W}(\alpha ) \end{array} \end{array}\}$$ (7)

$$\begin{array}{c} \left[\begin{array}{cc} m & o\\ o & m \end{array}\right]\left[\begin{array}{c} \varphi_{1}\\ \varphi_{2} \end{array}\right]=\left[\begin{array}{cc} C_{1}+C_{2} & -C_{2}\\ -C_{2} & C_{1}+C_{2} \end{array}\right]\left[\begin{array}{c} \varphi_{1}\\ \varphi_{2} \end{array}\right]\\ \varphi =-\varphi -\varphi^{3}\\ H=\frac{\varphi^{2}}{2}+\frac{\varphi^{2}}{2}+\frac{\varphi^{4}}{4} \end{array}\}$$ (8)

**Fig 4:**
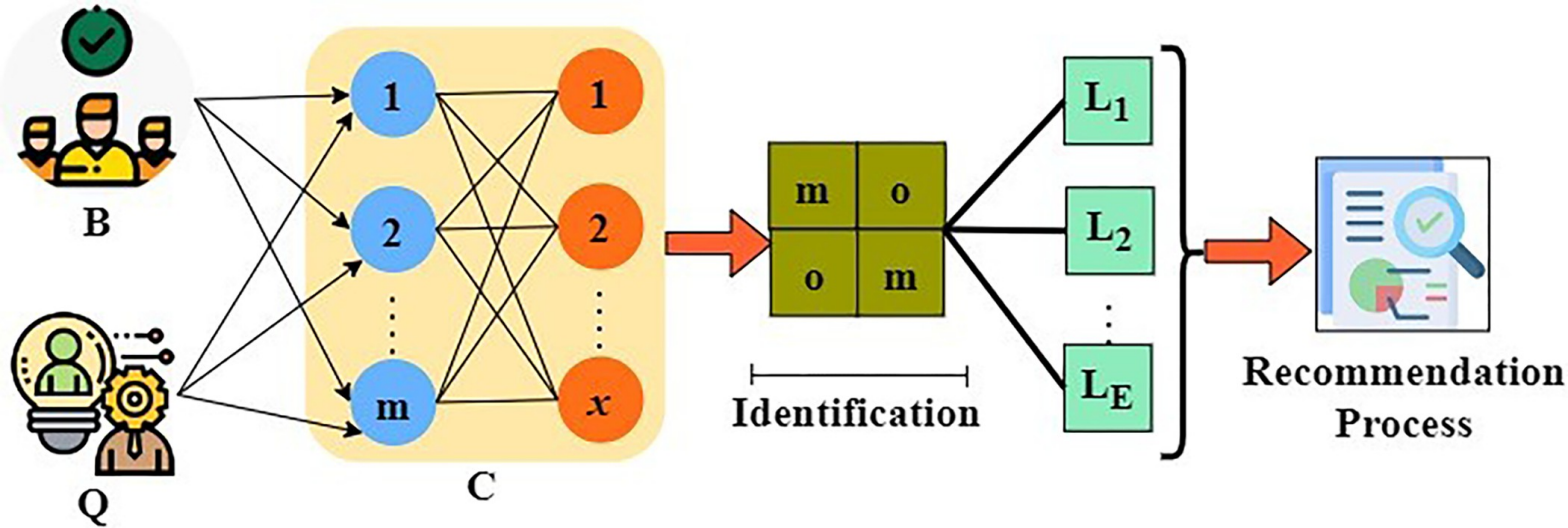
Output extraction.

From Fig 4, the notation *B* represents the process used to identify the management state, *Q* is denoted as the process of determining the skills utilized, *C* is denoted as the output of the assessment process, *m* is denoted as the data received from the previous process, *T* is denoted as the identification of the big data in the enterprise. The output extraction using the TL is portrayed in Fig 4.

In the second *C* process, the previous output *B* and new *Q* from (*α*−*W*) are used for state verification. The $\frac{\partial L}{\partial L_{E}}$ is performed for verifying (*m or o*) until the final recommendation is provided. This recommendation is either a position swapping or skill development. These two recommendations are intended for sustainable enterprise development (Fig 4). Now, the output of the placement of the management state and the skill utilization is interdependent to find out the achievement of the enterprise so far by using the transfer learning technique. The output denotes whether it has any losses or gains compared to the previous processes. If there is any loss in the identification of the outcome, then the recommendations will be given to enhance the enterprise with the precise management state. The output will be identified by using the outputs of the management state and the usage of the skills by the enterprise resources during the assessment process. From the assessment process results, the transfer learning is trained to identify the precise management state and the skills utilized by the management to produce effective outcomes. The profit and the loss of the enterprise resources will be mentioned in the outcome of the process. Thus, further steps will be taken to enhance the enterprises’ procedures in accomplishing effective achievements. The process of determining the outputs with the help of the placement and the skill management results are explained by the following Eqs (9) and (10) given below:

$$\begin{array}{c} \frac{\partial^{2}\varphi }{\partial x^{2}}+\frac{\partial^{2}\varphi }{\partial y^{2}}=\emptyset (x,y)\\ \varphi =\frac{1}{4}\sum_{C=1}^{4}\left(-1{)}^{C+1}2C(\pi x)(C\pi y\right)\\ \varphi (x,y)=\frac{-1}{2(C\pi {)}^{2}}\emptyset (C\pi x)\sigma (C\pi y) \end{array}\}$$ (9)

$$\begin{array}{c} PH\frac{\partial }{\partial \alpha }\varphi (x,\alpha )=\left[\frac{{-H}^{2}}{2m}\frac{\partial^{2}}{\partial x^{2}}+H(x)\right]\varphi (x,\alpha )\\ \frac{\partial }{\partial t}\left[\begin{array}{c} \varphi_{L}\\ \varphi_{I} \end{array}\right]=\left[\begin{array}{cc} 0 & \frac{-H}{2m}\\ \frac{H}{2m} & 0 \end{array}\right]\frac{\partial^{2}}{\partial x^{2}}\left[\begin{array}{c} \varphi_{L}\\ \varphi_{I} \end{array}\right]\\ \left[\begin{array}{c} \varphi_{L}\\ \varphi_{I} \end{array}\right]=\left[\begin{array}{cc} H & 0\\ 0 & H \end{array}\right]\left[\begin{array}{c} W_{L}\\ W_{H} \end{array}\right] \end{array}\}$$ (10)

Where *P* is denoted as the process of identifying the outputs of the enterprise resources, ∅ is denoted as the output of the placement of the state identification process, *y* is denoted as the output of the skill utilization by the enterprise management. Now if there is any loss in the output, then the recommendations will be given to the development process of the enterprises. The transfer learning is trained using the previously known gained enterprise achievement depending on the important enhancements. In this development procedure, the assessment performance will be increased based on the position of the resources. In this process, transfer learning assessment and the placement of the management state are very important. The enterprise development process, with the help of the recommendations given from the output, is explained by the following Eqs (11) and (12) given below:

$$\begin{array}{c} \Delta (x,o)=\frac{1}{\sigma^{\frac{1}{4}}\sqrt{\pi }}e^{\frac{-(x-x_{0}{)}^{2}}{\left(2\pi^{2}\right)}}+i\frac{x}{H}\\ \Delta (x,\alpha )=\frac{e^{\frac{-(x-(x_{0}+\frac{Q_{0}\alpha }{m}{)}^{2}}{2\pi^{2}(1+\frac{iH}{m\pi^{2}})}e^{i}\frac{\left(Qx-\alpha \right)}{H}}}{\sigma^{\frac{1}{4}}\sqrt{\pi (1+\frac{H\alpha }{m\pi^{2}})}} \end{array}\}$$ (11)

$$\Psi_{W}=(A_{H}^{T}A_{H}+H_{O}^{T}H_{O}+H_{x}^{T}x+H_{x}^{T}H_{x}{)}^{-1}(H_{O}^{T}\varphi_{O})$$ (12)

**Fig 5:**
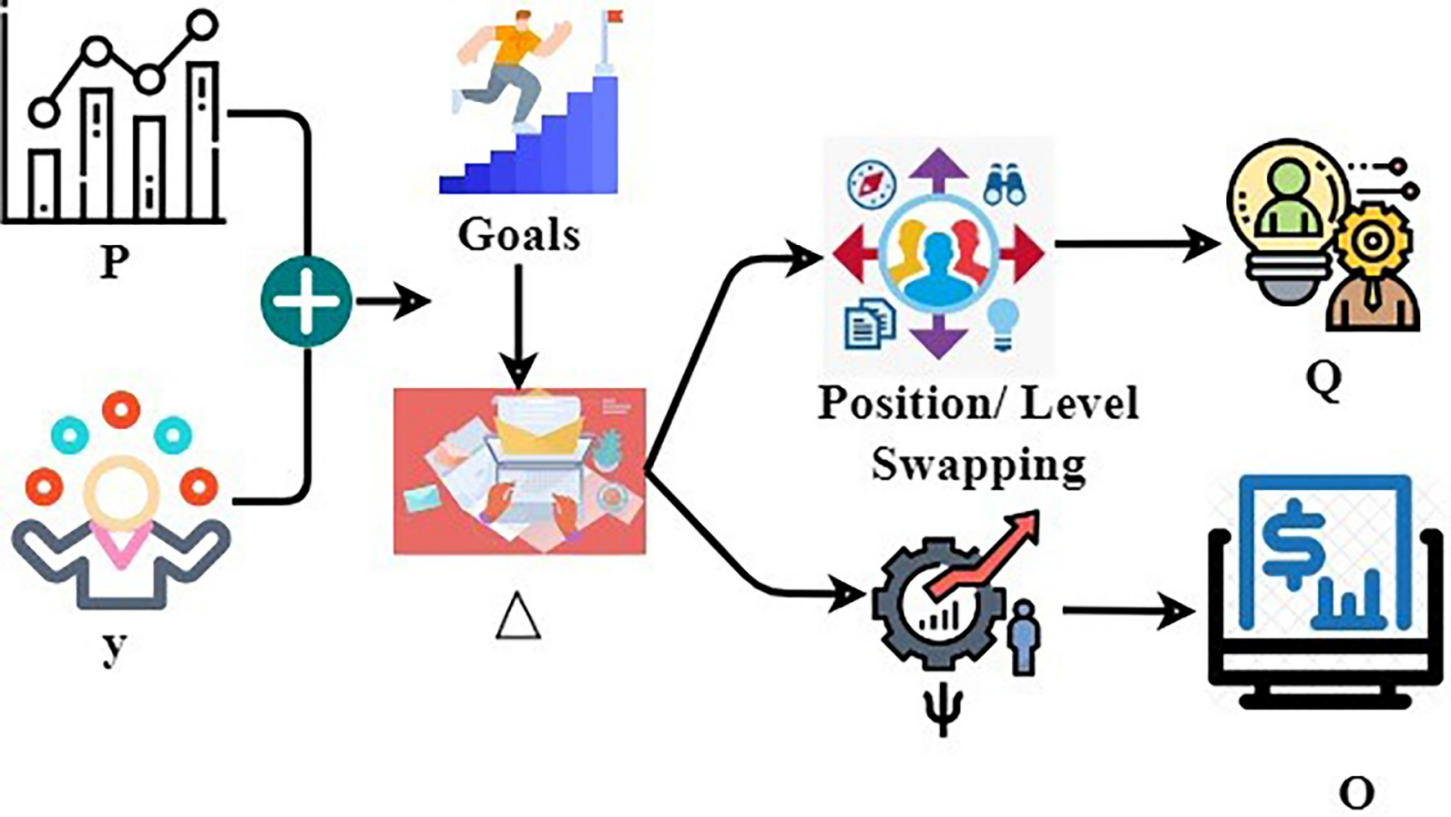
Recommendation process.

Where Δ is denoted as the recommendations given, Ψ is denoted as the process of developing the enterprises, *O* is denoted as the output after the recommendation. The recommendation process is presented in Fig 5.

In Fig 5, the input *P* and *y* given to managing the *O* for enterprise development is preferred from the *C* and *W*. It is required for goal development/ achievement from the enterprise end and therefore Δ is the final o for ensuring better sustainability (Fig 5). The assessment is performed for initial *L* and *Q*; the development in *L* is also identified using *T* (either o or m). This scheme is authentic in exchanging resources based on skills and enterprise claims before alleviating from the enterprise position /level swapping. Ψ is denoted as the process of developing the enterprises. Finally, the process of skills utilized is identified from *Q* and recommends the output for the enterprise. The enterprise management is developed by the recommendations sent from the achievement of the process. The transfer learning technique is used in finding the placement of the management state and the skills utilized by them. The learning technique is made for the assessment process to determine the resources’ performance and the trial period’s efficiency. The learning assessment and the management placement significantly helped develop the enterprises.

## 4. Discussion

**Fig 6:**
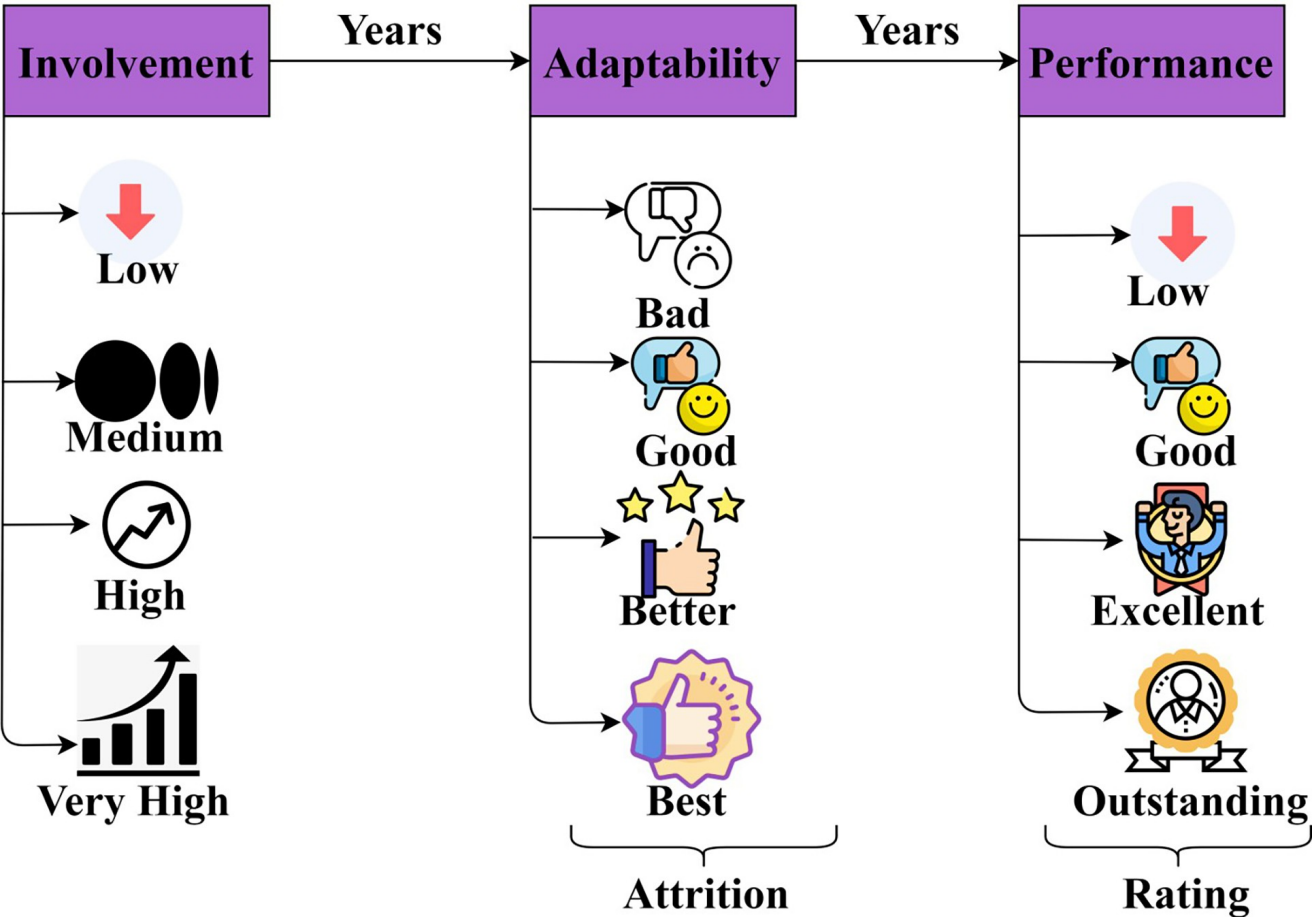
Sub-features representation.

The analysis is first presented by considering the input from https://www.kaggle.com/datasets/pavansubhasht/ibm-hr-analytics-attrition-dataset). This input is observed from IBM’s HR analytics for employee sustainability and performance. The data set is classified into "education," "environment," and "life balance" feature. From this information, involvement, adaptability, and performance are considered for improving enterprise development. The dataset contains an attribute named involvement, the factor relevant to Job Involvement (JI) with low, medium, high, and very high variables. In addition, the rating given for performance is considered low, good, excellent, and outstanding. It specifies various attributes related to the job role, level, previous role, number of years worked, performance rating, years in a current role, job satisfaction, and work-life balance are considered for employee movement from one role to another. The above information is utilized to investigate the performance analysis of the proposed system in terms of various performance metrics and increase the organization’s productivity based on the timeliness of work and ability. The additional (sub-features) are represented in Fig 6.

**Fig 7:**
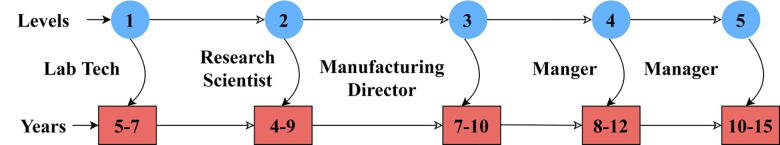
Different levels and experience years.

The sub-features directly impact the HRM based on enterprise improvement and staff management. Different skills are analyzed considering their performance from low to prompt level. This data presents five levels that consider different attrition and performance of the human resources. In Fig 7, the different levels and their skills considered are presented along with the considered years.

**Fig 8:**
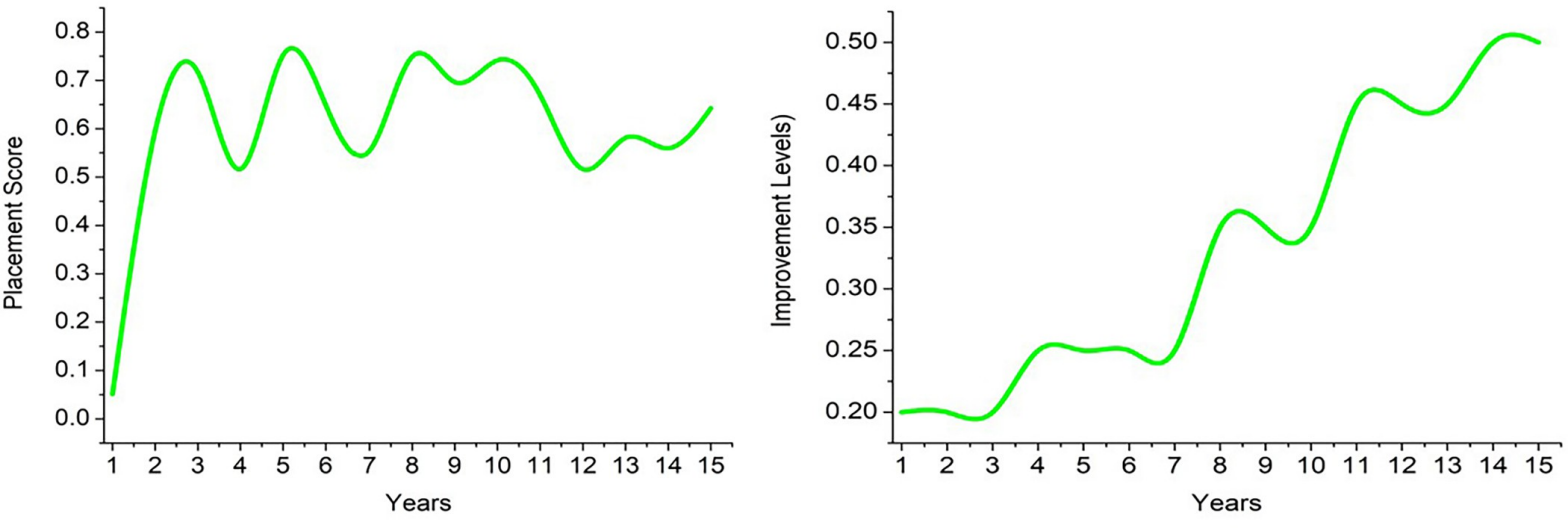
*W* ∀ placement and improvement (levels).

Based on the data represented above, satisfaction is considered across different resources. The swapping of positions occurs progressively based on the considered years. However, attrition decides the further progression based on the individual’s performance. First, the *W* for placement and improvement (levels) is analyzed in Fig 8.

**Fig 9:**
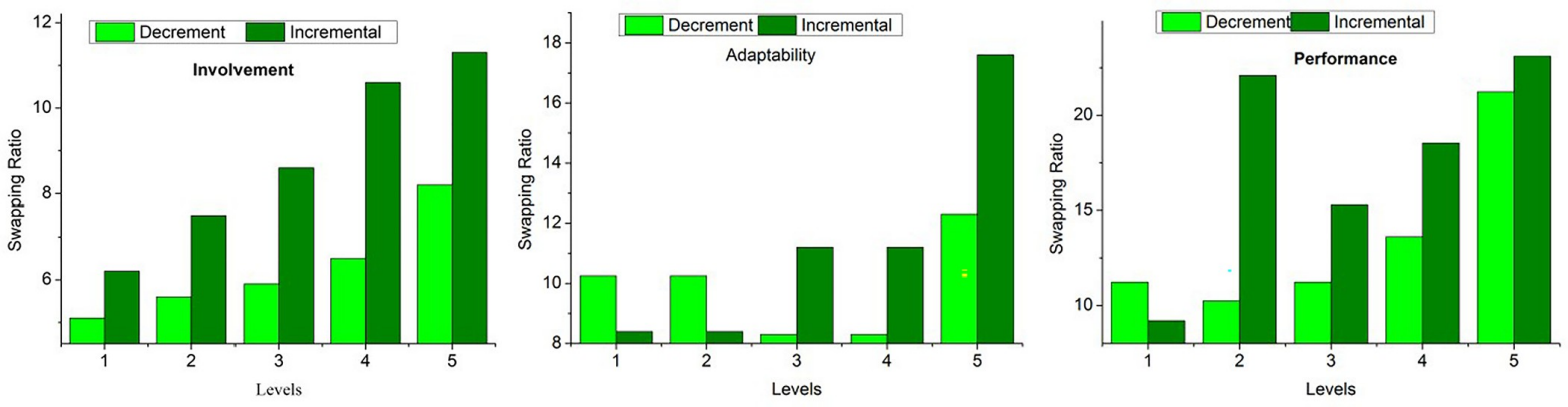
Swapping analyses for 3 factors.

The analysis in Fig 8 shows the cumulative placement and level improvement over 15 years. It refers to the overall resource count and their level of improvement based on performance and enterprise development. In the varying analysis, it is seen that the placement is high than level improvements based on *π* and *Q*∈*L*. The skill development and performance improvements are used for identifying the swapping for varied improvements. Based on the three factors presented in Fig 6, the swapping rate is analyzed in Fig 9. This includes incremental and decrement levels (Swapping).

Compared to adaptability and involvement, the swapping ratio is higher for the performance feature. The transfer learning updates the training inputs from (*L*, *V*) to (*B*, *Q*) from the previous intervals. Such updates are required to prevent earlier resource mitigation without providing a chance. Therefore, the change in $\left[\begin{array}{cc} \begin{array}{c} m\\ o \end{array} & \begin{array}{c} o\\ m \end{array} \end{array}\right]$ alternates *ψ* for maximum *y*, before mitigation.

Besides the above discussion, the following section provides a comparative analysis using the metrics placement success rate, resource swapping, mitigation, decision time, and data analysis. The operations vary between 20 and 360 for a maximum of 12 operating levels. The methods RFNE [26], EHRM-FPGA [22], and EHRM-DSS [23] are considered alongside the proposed PRMS in the comparative analysis.

### 4.1 Placement success rate

**Fig 10:**
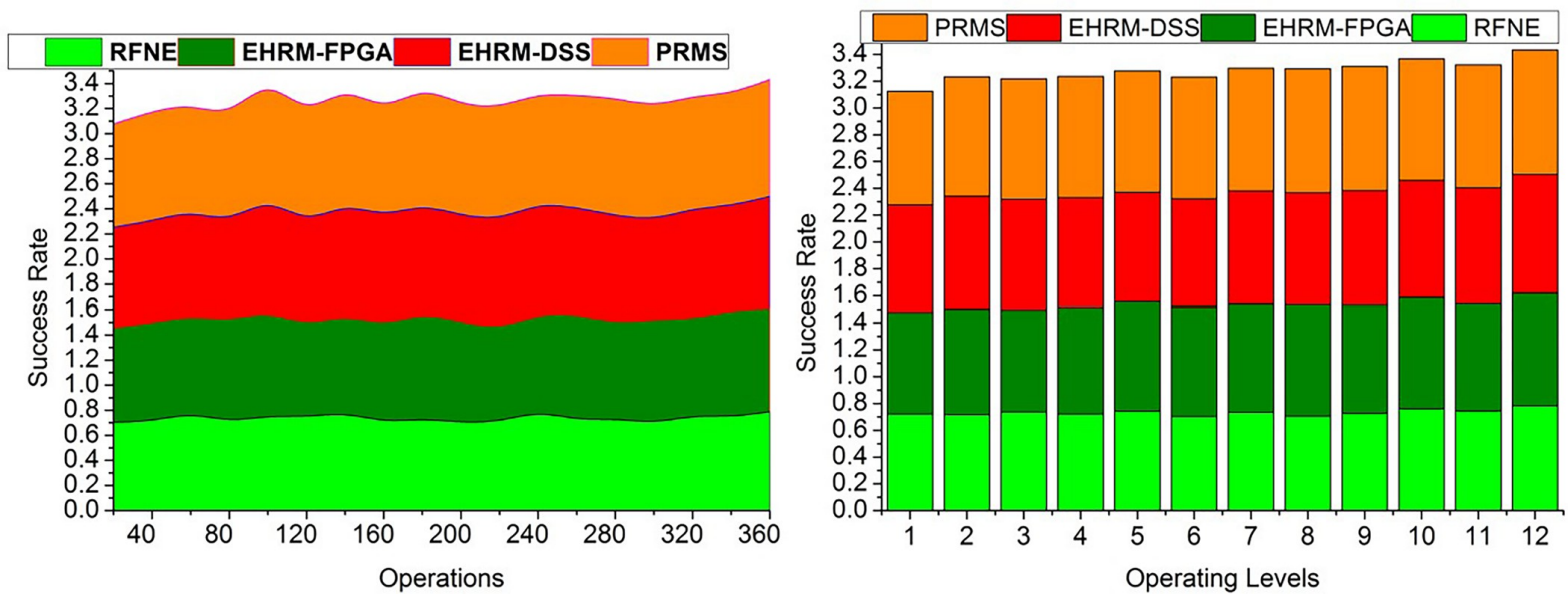
Placement success rate.

The placement success rate is productive in this method by using the transfer learning technique for the production of the effective outcome of the enterprise. The placement success rate depends on many factors, including the resource assessment’s accuracy at various operating levels, the dynamic nature of enterprise operations, and its accomplishments. Here, the management state is found, thus determining skill utilization. Then these outcomes will be interconnected to verify the enterprise’s best achievement. The transfer learning is trained well to identify the precise management state and the output of the assessment process to help determine the enterprise’s placement. From the outcome of the assessment process, by using the data acquired, the identification of the placement of the enterprise management and the utilization of the skill can be determined. The mentioned factors, such as perfect enterprise procedures and financial outputs, are determined first in a trial period by resource placement. The transfer learning is trained to identify a correct management state depending on the trial assessment outputs. Finally, the state of the management is used to determine the process level and whether it can deliver on time (Fig 10).

In Fig 10, the enterprise’s placement success rate is depicted based on operations completion level on time and various operating levels up to 12. The graph shows that the current work outperforms the existing algorithms RFNA, EHRM-FPGA, and EHRM-DSS.

### 4.2 Resource swapping

**Fig 11:**
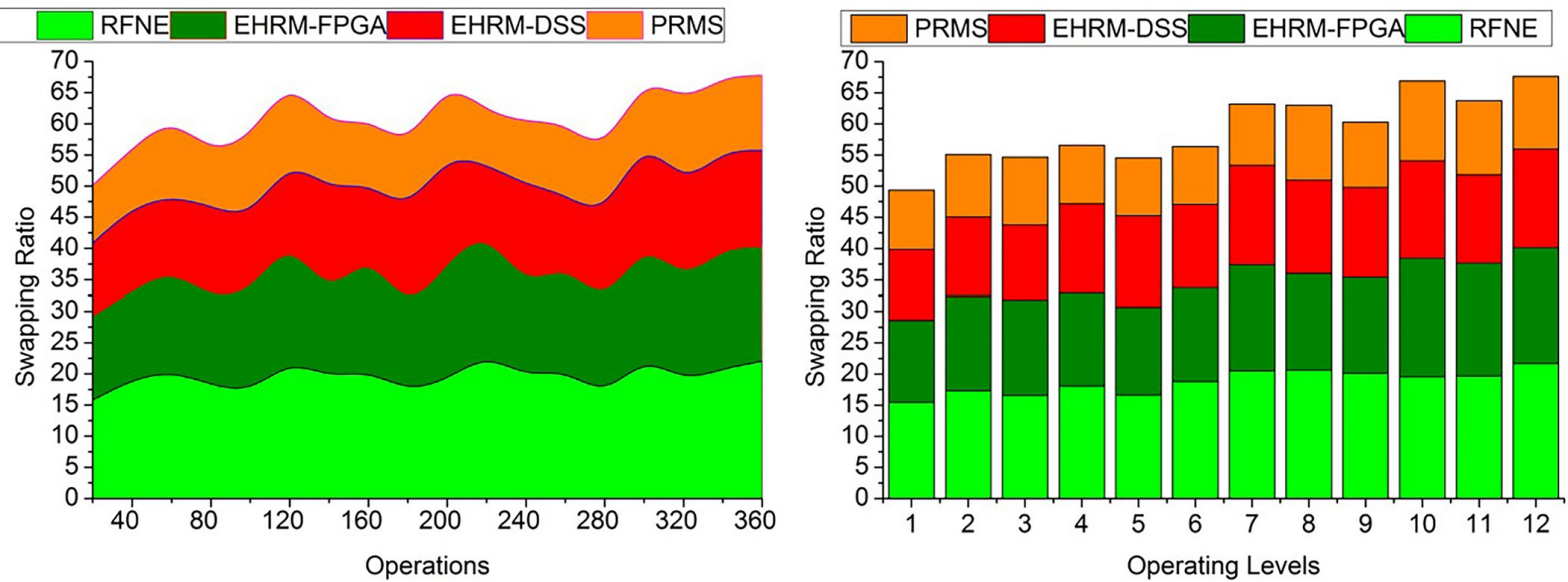
Resource swapping.

Resource swapping is reallocating or reassigning organizational resources in response to shifting demands and assignment outcomes. Employees may be moved across teams, departments, or projects based on skills to maximize resource usage and boost the operational effectiveness of enterprises. The resource swapping is less in this method by using the outcome of the assessment process through the transfer learning technique. Human resource management manages the employees and the operation deliverance at its correct time. The data which is acquired from human resource management will be helpful in the assessment process. Thus, the output of the performance of the resources will be gainful in achieving the best enterprise outcomes. From resource management, the position and the skills of the resources can be identified. Then with the data acquired, the assessment process is done by using transfer learning. The swapping of resources is less during the production of the enterprise outcome. Transfer learning is trained well for the identification of the placement of the management state. Also, it will be helpful in the enterprise development with the recommendations provided from the achievement. In that enterprise development process, the management position and the transfer learning assessment will be significantly important (Fig 11).

### 4.3 Resource mitigation

**Fig 12:**
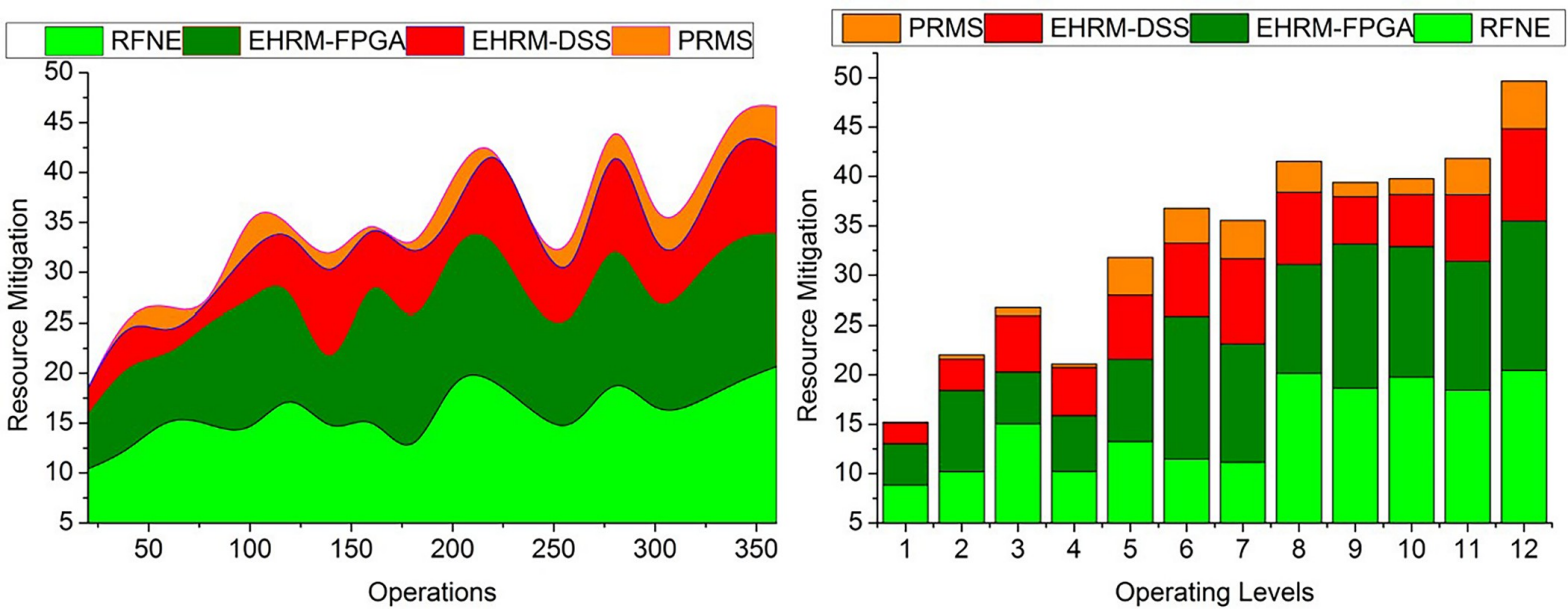
Resource mitigation.

Resource mitigation is dealing with or overcoming problems or difficulties relating to resources within a business environment. Mitigation often entails formulating and implementing plans to lessen or resolve resource-related issues that can impede production or operational effectiveness. Resource mitigation method efficiency depends on how well they fit organizational needs and how well a business can react to changing conditions are just a few of the aspects that will affect how successful they are. The mitigation of the resources is tendered less in this method by using the data from human resource management. Skills of the resources are denoted as the skills acquired by the resources which lead to the maximum outcomes for the enterprises. The alleviation of the resources will lessen with the prompt state of the management and the data from the previous output. Transfer learning is used to conduct the assessment process from the position output and skills determination procedure. The enterprise resources’ performance and the trial period’s efficiency will be detected from the assessment process. The seriousness of the resources obtained from the enterprises is less with the non-swappable management state and the correct determination of the resource skill utilization during the production of the efficiency outcome. It improves the enterprise’s productivity with a precise management state and a better assessment rate. By this, the mitigation of the resources is less with the help of the transfer learning technique (Fig 12).

### 4.4. Decision time

**Fig 13:**
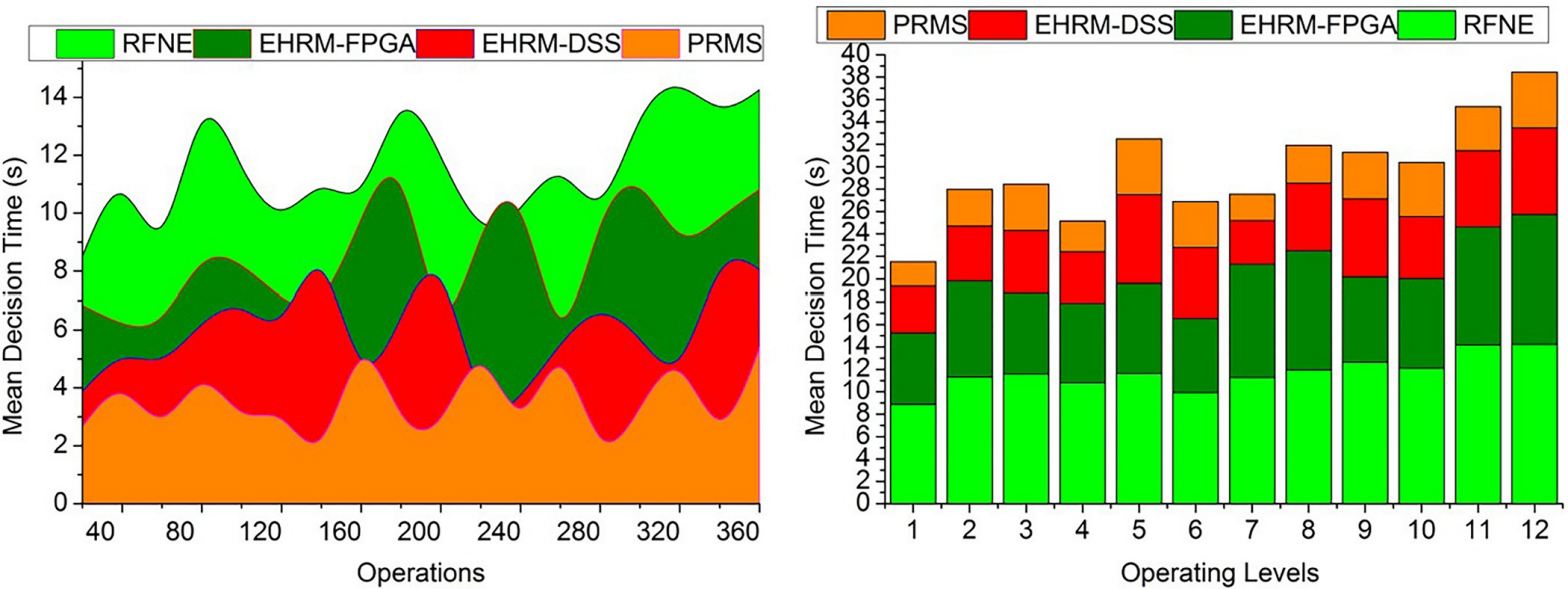
Decision time.

This research investigates the relationship between rationality in making decisions and the depth to which a business adopts an enterprise resource planning system. The amount of time it takes to make decisions about resource management inside an organization is called decision time. The complexity of the decision-making, the accessibility and reliability of the information, the participation of stakeholders, and internal organizational decision-making processes can all affect how long it takes to make resource management decisions. System utilization and user satisfaction are analyzed as potential mediators between the depth of adoption and the effectiveness of an organization. Analysis and optimization techniques may be used to find the best action by investigating several choices and weighing their relative merits. The time taken to make the decision is less with the help of the transfer learning technique and with the output of the better assessment performance of the enterprise resources. The achievement of the enterprises will be detected from the output of the placement and the skill utilization by the learning technique. The performance of the enterprise management is used to determine the skills they gained in the trial period. From the output, the recommendations will be given to developing the enterprise along with the increment of the assessment by altering the position of the assessment. The transfer learning assessment and the precise placement of the resource management are very important in developing the enterprise. It can improve the enterprise and the performance of the resources in the assessment procedure. From this, the decision will be taken, and also, by changing the position of the resources, the performance can be enhanced during the production of effective enterprise achievement. Depending on the important enhancements, the transfer learning is trained using the previously known gained enterprise achievement (Fig 13).

### 4.5. Data analysis

During the trial phase, data analysis can be utilized to assess the abilities, credentials, and productivity of the resources. Data analysis is an excellent way to evaluate the efficiency of resources at various operational levels. Organizations may make decisions based on data to improve and optimize the PRMS over time by examining performance, feedback, and results data.

**Fig 14:**
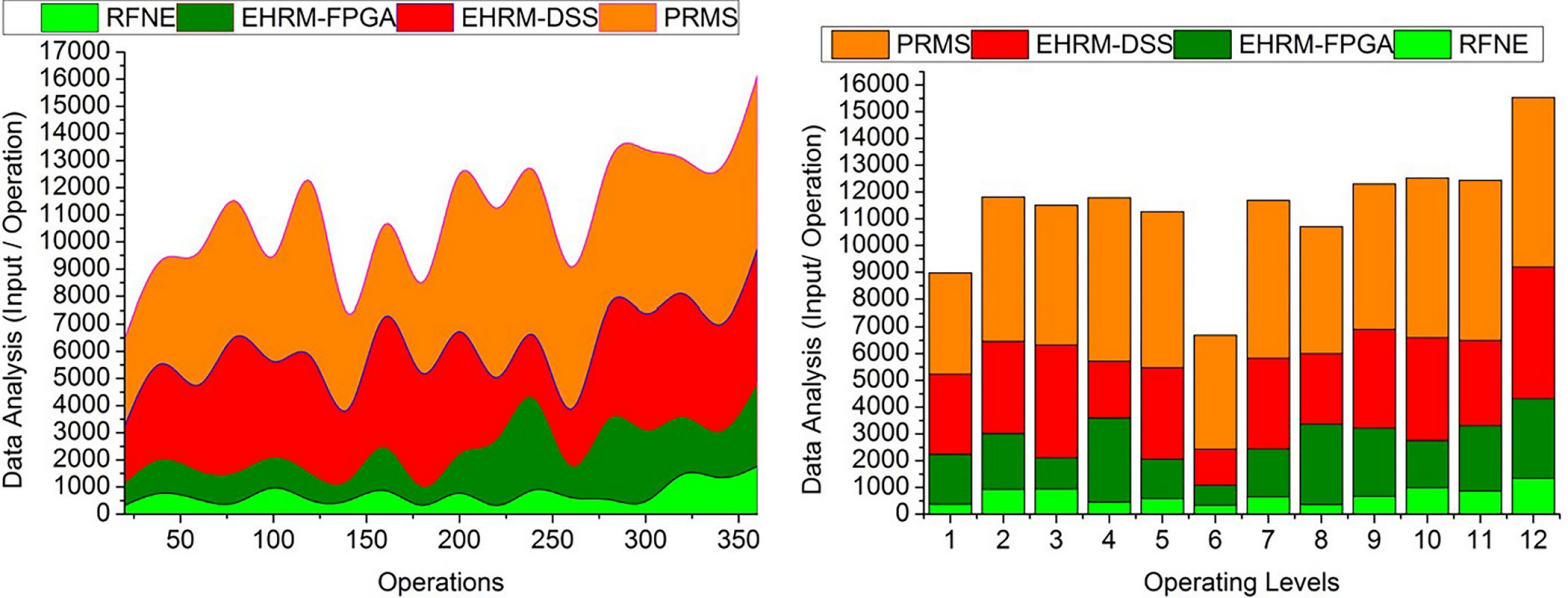
Data analysis.

The data analysis is productive in this method with the help of the learning technique in determining the precise enterprise management state (Fig 14**)**. The HR designation and the skills determination identify the skills executed by the HR, leading to the effective maximum outputs. The assessment done from the output of the data gained is used to determine the positional/managerial placement of resources by pursuing their professional skills. The above-stated features, apt enterprise operations, and financial outcomes are verified first in an experimental manner by resource placement. Then, the procedure is processed with the above-stated data acquired from resource management. After the trial period, the available resources are identified for their skills and work perfectness. HR efficiency is estimated with the output of the assessment process and the employees’ performance. The output of the assessment is used to determine the placement of the resource management with the transfer learning technique. Placement output and skill utilization output are interconnected to provide an efficient outcome for enterprises. The comparative analysis results are tabulated in Tables 2 and 3 for operations and operating levels.

**Table 2:** Comparative analysis for operations.

Metrics	RFNE	EHRM-FPGA	EHRM-DSS	PRMS
Success Rate	0.788	0.825	0.886	0.9328
Swapping Ratio	22.06	18.19	15.46	12.052
Resource Mitigation	20.67	13.32	8.513	4.098
Mean Decision Time (s)	14.26	10.81	8.05	5.414
Data Analysis (Input/ Operation)	1759	3099	4878	6387

**Observations:** The proposed scheme is responsible for increasing the success rate and data analysis by 9.98% and 8.2%, respectively. This scheme also reduces the swapping ratio and resource mitigation and means decision time by 6.52%, 13.84%, and 8.49%.

**Table 3:** Comparative analysis for operating levels.

Metrics	RFNE	EHRM-FPGA	EHRM-DSS	PRMS
Success Rate	0.783	0.836	0.884	0.9302
Swapping Ratio	21.72	18.41	15.82	11.658
Resource Mitigation	20.47	15.01	9.361	4.815
Mean Decision Time (s)	14.24	11.49	7.74	4.976
Data Analysis (Input/ Operation)	1341	2978	4881	6335

**Observations:** The proposed PRMS scheme is responsible for increasing the success rate and data analysis by 9.59% and 8.6%, respectively. This scheme also reduces the swapping ratio, resource mitigation, and means decision time by 6.7%, 11.3%, and 9.2% compared to other existing methods such as RFNE, EHRM-FPGA, and EHRM-DSS. Successful supply chain management is challenging due to the interconnected nature of the many activities and organizations that comprise a complete supply chain cycle. As a result, it becomes very difficult for firms to document any procedures. With the help of our suggested technique and Big Data, businesses may get a comprehensive look into their whole supply chain, including all of their operations and assets. Product flows, and movements are tracked and simplified so that firms can keep tabs on every process step. It makes it considerably simpler for companies to develop a long-term strategy.

### 4.6. Resource Utilization Efficiency(RUE) analysis

RUE assesses how efficiently resources are used within an enterprise organization to complete responsibilities or jobs, contribute to productivity in general, and produce positive financial results. RUE is a metric that measures how well available resources are used within an organization. It evaluates the effectiveness of resource management and allocation in achieving targeted business results. By contrasting the actual production or productivity attained with the prospective output that could be attained with optimal resource utilization, RUE may be estimated. The RUE formula can be calculated using Eq 13 as follows:

RUE=(actualoutputMaximumoutput)×100 (13)

While potential output reflects the highest level of productivity or results that could be reached under optimal resource utilization conditions, the actual output represents the productivity or business results obtained with the resources allotted.

**Fig 15:**
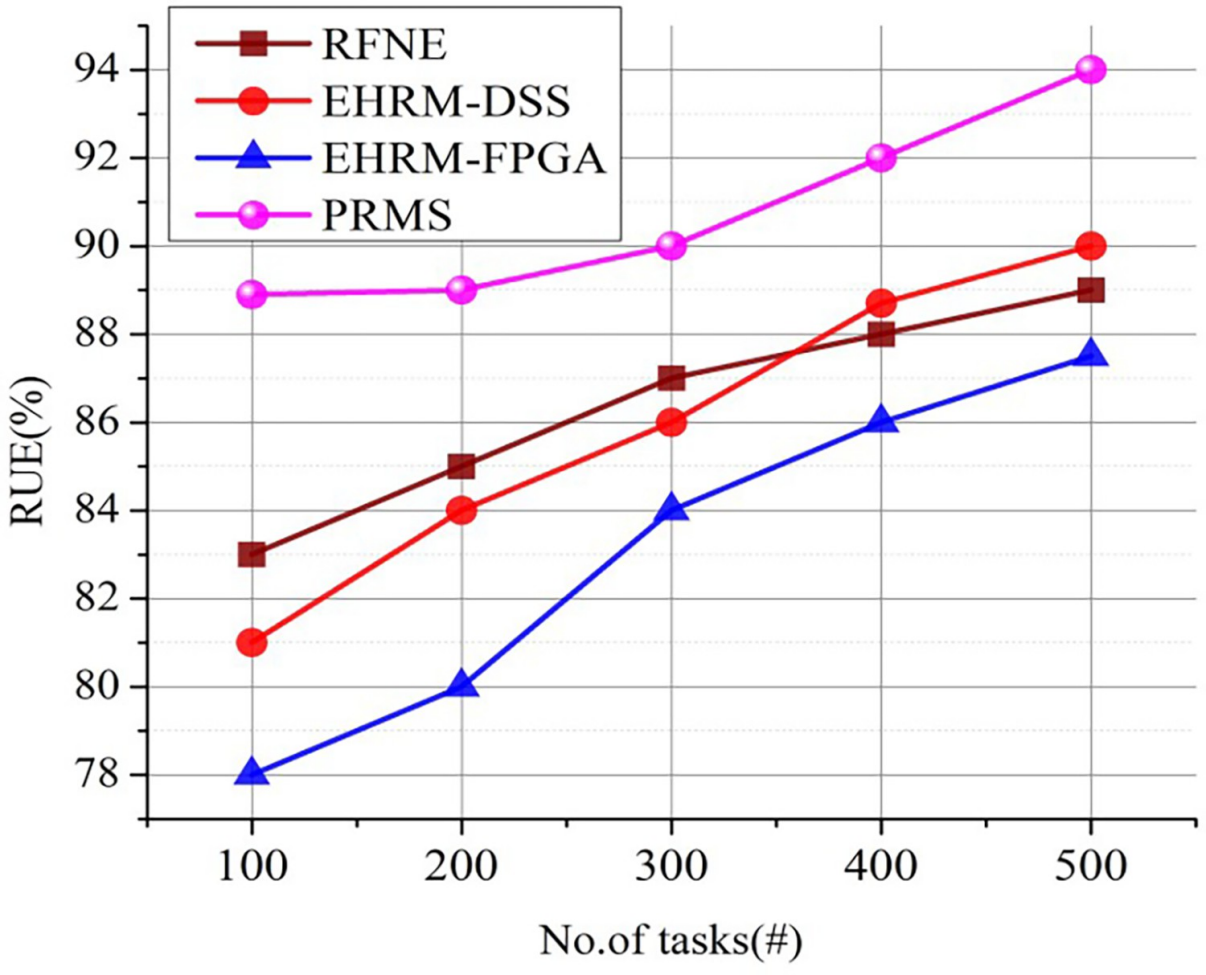
RUE analysis.

Fig 15. illustrates the enterprise outcome based on some tasks a resource completes, and its results are compared with existing approaches. The proposed system outperforms the existing one in terms of higher RUE; thus, it is recommended to use in real-time business enterprises. A greater RUE % denotes more effective resource use, demonstrating that resources are strategically put and used to their fullest potential to maximize productivity and financial results. A lower RUE %, on the other hand, denotes inefficiencies in resource deployment and utilization, suggesting space for development. Businesses can evaluate the efficacy of PRMS and pinpoint areas for resource management improvement by tracking RUE over time. Enterprises can see trends, assess the effects of resource placement choices, and make modifications to improve resource utilization and overall performance by tracking RUE. Enhancing RUE can result in greater production, cost reductions, and better financial outcomes.

## 5. Conclusion

### Research findings

The Placement-Assisted Resource Management Scheme (PRMS) shows how big data analytics may optimize resource allocation and boost company efficiency. The PRMS uses transfer learning to detect accurate management levels and skill use, enabling efficient resource deployment. Practical RUE evaluation is crucial to corporate success, according to the study. The proposed system PRMS outperforms RFNE, EHRM-FPGA, and EHRM-DSS in multiple performance indicators at two different levels of operations and operating levels, with a success rate of 0.9328% and 0.9302%, minimal swapping ratios of 12.052% and 11.658%, smaller resource mitigation ratios of 4.098% and 4.815%, mean decision times of 5.414s and 4.976s, and data analysis counts of 6387 and 6335 Success and data analysis increase by 9.98% and 8.2%, respectively, with the proposed strategy. This technique cuts the switching ratio, resource mitigation, and decision time by 6.52%, 13.84%, and 8.49%, respectively, and applies to various operations.

### Theoretical contribution

The data-driven resource allocation approach introduced in the study advances human resource management theory. Transfer learning and big data analytics give the PRMS a theoretical framework for improving HR designation, skills assessment, and enterprise management state assessment. The theoretical basis of RUE analysis helps evaluate resource management efficacy. Resource switching and talent utilization/update for enterprise-oriented outcomes using exact management status are proposed. The PRMS includes the latest successful enterprise operation for training and profitably sustaining/leveraging enterprise operations.

### Practical contribution

The PRMS helps firms optimize resource use and operational efficiency. HR designations and skills execution data can inform business resource placement and skill use. Transfer learning allows firms to maximize outputs and profits through resource management. Managers who promote skill development and ongoing learning can match employees’ skills to job requirements.

### Potential limitation

The research limitation lies in integrating analytics for big data and methods for transfer learning into existing human resource management systems, which may present specific difficulties. Additionally, the efficiency of the PRMS may differ depending on the business size, the sector in which it operates, and the technological infrastructure in place.

### Future research

Future empirical studies could confirm the PRMS across diverse organizational situations. Exploring the PRMS’s industry scalability and implementation issues may improve it. The PRMS’s long-term benefits on company productivity and satisfaction among workers may encourage the utilization of resources and optimization study through machine learning and predictive analytics.
